# Report of the Superintendents of the Poor of Erie County, for the Year Ending Oct. 1, 1849

**Published:** 1849-12

**Authors:** 


### EDITORIAL DEPARTMENT.

*Report of the Superintendents of the Poor of Erie County, for the year ending Oct.* 1, 1849.

We had been looking, for some time, for the publication of this Report, and began to think we should be obliged to consult it in manuscript, in the county archives, when, at length, it has made its appearance in one of the city papers. Heretofore it has been customary to publish this report during the annual meeting of the Board of Supervisors, in October, but, for some reason, the present year, it has been withheld from the public until the second meeting of the Board, after the election of county and state officers.

The amount of expenditures for the support of the county poor during the last year, it appears, has exceeded, considerably, that of the previous year. But this is a matter which it does not belong to us here to discuss. We leave it for the Superintendents and Supervisors, whose function it is to consider the subject of pauper relief in its pecuniary aspect, and, as it would seem, in this aspect alone—for the idea of providing comfortable accommodations for the indigent sick, and of attempting to diminish the enormous rate of mortality among this class, has not seemed, hitherto, to enter into their deliberations. Judging from the absence of both actions and words, the claims of humanity with respect to those for whose lives and health these public functionaries are in some degree responsible, have, as yet, scarcely had the benefit of a moment's reflection. The sole end would appear to be to diminish the poor tax. Would that it might be abolished altogether! Philanthropy, and true charity, might then have some chance for admission into provisions for the relief of the sick poor. In thus referring to the county Supervisors, we would be understood to speak of them collectively. There have been, and are individuals in the Board, to whom we would not impute that indifference to all else beside dollars and cents, which we cannot but think attaches to the body corporate; and it is, unfortunately, often the case that persons would not incur the responsibility of acts, or omissions, singly, as individuals, which they are willing should be divided among each other in an official aggregation. We will simply remark, with respect to the financial part of the report before us, that the increase of expenditure over that of the year previous, is attributed to the arrival at our ports of an increased number of foreign immigrants. "This (in the opinion of the Superintendents, quoting the peculiar phraseology of the report), might be a sufficient reason for our great growth in this branch of business," i. e., pauper business! This, however, would hardly seem to afford an adequate explanation, for, on comparing the number relieved at the Poor House during the past year, with that of the previous year, we find the former to exceed the latter by only thirty-one persons, while the relative excess of expenditure is over three thousand dollars. This is a pretty liberal allowance for the maintenance of thirty-one additional paupers during the time they remained at the Poor House! But the data for determining precisely what the additional expense would be expected to be, are at hand. The average price of board, per week, for each pauper, is stated to be a fraction over 70 cents. Dividing the whole number of weeks' board (which are given in the report) with the whole number of inmates, gives the average time each pauper remains in the Poor House, and, the result is, nine weeks. Thirty one more paupers at the Poor House than were supported there during the previous year, should then have cost the county about three hundred dollars, instead of three thousand. The increase of emigration, hence, is not sufficient to account for twenty-seven hundred dollars of the increased expenditure at the Poor House. This may be susceptible of satisfactory explanation, but, as we have said, a financial criticism of the report is not our present business. We proceed to quote some other facts embraced in the Report.

The whole number of persons committed during the year is 1,015. Of this number, 151 were vagrants, 35 were insane, and 1 an idiot. There were 25 births at the Poor House during the year—a pretty good basis for a Lying-in Asylum! The number of deaths at the Poor House during the year, was 124. Of the persons committed to the Poor House during the year, 152 were native citizens, and 863 were foreigners. The number of persons receiving out-door, or temporary relief, was 7,376. Number of deaths among the latter, 499. The rate of mortality, it will be perceived, is somewhat less than for the previous year. (See Buffalo Medical Journal, number for Dec., 1848). We make the ratio a fraction less than 1 to every 8 of the whole number of persons committed. For the previous year the ratio was as 1 to about every 6. We know not to what to attribute this improvement, which is the more worthy of note, because the epidemic cholera prevailed, to some extent, among the inmates of the establishment. It is, perhaps, fair to attribute the merit of reducing the rate of mortality, to the keeper and physician, Dr. J. B. Pride. The plan of uniting these two offices was entered upon during the last year, and possesses many obvious recommendations. By this arrangement, it is to be presumed, are secured better regulations, as respects hygiene, and prevention of disease, and, at the same time, more efficient medical services, from the fact of the physician being always on the spot.

Still, the mortality is enormous, arising from causes which no care or skill in the physician and keeper can control, and which the Superintendents, in their report, admit, when they speak of the "inadequately and ill adaptedness" of the present county buildings, recommending, also, as they do, the purchase of a farm, and the erection of more suitable buildings. It would be unjust to the Superintendents not to add, that this recommendation is made not only on account of "every consideration of interest," but "humanity and duty to the poor." We are right glad to see such an expression as this last, in a report from that quarter. This is a decided improvement upon the report of last year.

The rate of mortality which has been given, does not express the proportion of deaths to the number of cases of sickness, for, it is to be considered, that all persons committed to the Poor House are not suffering from disease. We find, by the report, that 151 were committed as vagrants. Probably few, if any of these were sick, or, at all events, it would be a liberal admission to concede that the proportion of vagrants, sick when committed, or affected by disease afterward, would not more than balance the proportion of paupers committed for poverty alone, without sickness. Deducting, then, the 151 vagrants, together with the 35 insane persons, and the 1 idiot, the remaining number will be 824. Dividing this by the number of deaths, and the mortality will be a fraction less than 1 to every 6. This should be taken as the rate when comparison is made of the Poor House, with Hospitals, and other institutions receiving sick persons only. We know of no Hospital in the country where the rate of mortality approximates to that at the Poor House in this county. Institutions receiving precisely the same kind of patients, such as the Bellevue Alms House Hospital, N. Y., the Quarantine Hospital, Staten Island, N. Y., present a proportion of fatal cases in the one, nearly one-third, and, in the other, three-fourths, less than at the Erie Co. Poor House! This we showed by comparison of statistics in an article on this subject in the Dec. number of this Journal, 1848, to which the reader is referred. At the Hospital of the Sisters of Charity, in this city, the ratio of mortality for the six months ending Sept., 1st, 1849, exclusive of cases of epidemic cholera, was one to a fraction less than 15. Including the 134 cases of epidemic cholera received during the period referred to, the proportionate mortality of the sick was but a fraction greater than that which we have estimated at the Poor House, for the past year, albeit, the latter exhibits an improvement upon the previous year. Now what is the inference from these facts? Why, that, at the lowest estimate, one-half of those who have died at the Poor House, during the past year, might have been saved, had they enjoyed the same advantages which are extended to the sick poor in other institutions. We would urgently invite the attention of our readers residing in this city and county, to this startling conclusion. We would fain discover some way of escaping it, but the truth stands demonstrated by plain calculations from data that cannot be gainsaid. The "inflexibility of arithmetic" leaves no room for incredulity. A truth so lamentable should not be concealed. Let it be sounded in the ears of this community, that, of the sick persons which public charity assumed to provide for, at the Poor House, during the past year, at least *sixty-two* have been sacrificed to the want of needful Hospital advantages for recovery! We ask our medical readers in this city and county, not only to ponder well upon this reckless disregard of the claims of humanity, and indifference to the value of human life, but to bring the subject to the apprehension and serious consideration of their friends and neighbors. It is not a new subject to us. We have heretofore labored to direct public attention to it, and enlist that interest which it claims, but, we are free to confess, with discouraging success. Impressed, as we now are, more than ever, with its vast importance, we cannot resist a sense of duty which impels us to resume our humble efforts for the same end, and, if life and health are spared, it shall not be for the want of the exercise of the little ability God has given us, if facts and legitimate inferences relating to the subject of pauper relief in this county do not receive some measure of that appreciation which they deserve. May we not presume that our professional brethren will be ready to cooperate in such an undertaking?

Responsibility for the present destructive system of relief for the sick poor, (if we may use such an expression) is somewhere involved, since none can contend that the evils are irremediable. Where does this responsibility rest? We answer, first, that the Superintendents of the poor are bound to know the extent of the suffering, and loss of life incident to the existing state of the department of charity (charity!) under their administration. They have no right to be ignorant of the fact that the deaths at the Poor House might be reduced one-half by commodious buildings, suitable appliances, and proper nursing. Knowing this, as they should do, it is plainly their duty to do all in their power to effect the required improvements. If they have done all this, they are acquitted of all responsibility. Second, the Supervisors are bound to investigate the whole matter. They are not commissioned to legislate solely for the interests of the tax payer. They cannot escape before God, if before their constituents, the duty of providing in the best manner, for the safety and welfare of those whose lives are dependent on their deliberations. Has this duty been performed?

Finally, each individual member of this community is responsible, in proportion to his acquaintance with the merits of the subject, and his ability to exert an influence in behalf of those whose vital interests, in a literal sense, are to be effected by the success of his efforts.

The space to which we are limited, obliges us now to bring this article to a close, but with the intention of resuming our remarks (here, or elsewhere) at a future time. There are important facts embraced in the report of the Superintendents of the poor, inviting comment, to which no allusion has, as yet, been made. In the mean time, let the reader consider if there be any topic calling for public interest and action, which, at this time, possesses greater importance than this! Suppose the intelligence were to be communicated that a steamer had been wrecked, or burned, *sixty-two* persons having been unexpectedly called into eternity—what horror would be depicted upon every countenance! Is it less shocking that sixty-two persons have died by disease, when, by proper measures at our command, their lives might have been spared? The former is a fiction, the latter is an appalling fact. In the former case, no prudence, or foresight, perhaps, could have averted the calamity; in the latter case, human means were fully adequate to avert the evil. We commend these thoughts to the meditations of those who may honor this article with a perusal.

